# Perspectives on benchmarking foundation models for network biology

**DOI:** 10.1002/qub2.68

**Published:** 2024-07-11

**Authors:** Christina V. Theodoris

**Affiliations:** ^1^ Gladstone Institute of Cardiovascular Disease San Francisco California USA; ^2^ Gladstone Institute of Data Science and Biotechnology San Francisco California USA; ^3^ Department of Pediatrics University of California, San Francisco San Francisco California USA

**Keywords:** benchmarking strategy, foundation models, network biology, transfer learning

## Abstract

Transfer learning has revolutionized fields including natural language understanding and computer vision by leveraging large‐scale general datasets to pretrain models with foundational knowledge that can then be transferred to improve predictions in a vast range of downstream tasks. More recently, there has been a growth in the adoption of transfer learning approaches in biological fields, where models have been pretrained on massive amounts of biological data and employed to make predictions in a broad range of biological applications. However, unlike in natural language where humans are best suited to evaluate models given a clear understanding of the ground truth, biology presents the unique challenge of being in a setting where there are a plethora of unknowns while at the same time needing to abide by real‐world physical constraints. This perspective provides a discussion of some key points we should consider as a field in designing benchmarks for foundation models in network biology.

1

The machine learning approach of transfer learning involves leveraging large‐scale general datasets to pretrain models with a fundamental understanding of the learning domain that can then be democratized to a vast array of downstream tasks, enabling predictions with little or no task‐specific training data. This differs from standard approaches where task‐specific data is used to train a model using a task‐specific training objective to make predictions in that particular task. In this standard case, if you would like the model to make predictions in a new task, you would have to retrain it from scratch with new task‐specific data and a new task‐specific training objective, therefore not taking advantage of the large‐scale data available more broadly. In contrast, with transfer learning, the large‐scale pretraining phase imbues the model with foundational knowledge that can then be transferred to a multitude of diverse downstream applications. The foundation model can either be fine‐tuned toward a specific downstream application with limited task‐specific training data, or it can be used directly to make predictions in the downstream task via zero‐shot learning.

Transfer learning has revolutionized a broad range of informational fields, including natural language understanding [1, 2] and computer vision [3]. More recently, there has been a growth in the adoption of transfer learning approaches in biology. We are now at a critical threshold where we have sufficient biological measurements to pretrain models with a fundamental understanding of biological systems. Research groups have pretrained foundation models for structural, genomic, and network biology using massive amounts of biological data and demonstrated the value of such approaches in improving predictions in settings obstructed by limited data and accelerating biological discoveries [4, 5, 6, 7, 8, 9, 10, 11, 12, 13, 14, 15, 16, 17, 18, 19].

As we continue to expand our approaches using transfer learning to model biological systems, it is important that we engage in open community discussions on strategies for benchmarking these models. Benchmarking involves testing the performance of various models on a set of standardized tasks so they can be directly contrasted in a controlled manner. For example, Liu et al. recently tested multiple foundation models for network biology on a diverse array of downstream tasks [20]. Importantly, benchmarking is not merely about comparing models but about guiding the evolution of our modeling approaches toward achieving our ultimate biological goal. It is critical to develop benchmarks that focus our efforts on biological insights that are the ultimate goal of the models. Otherwise, we risk merely becoming very effective at developing models that perform very well at a task that is not actually our end goal. For example, if we optimize models to tightly cluster cell types for visualization but this deemphasizes the models’ understanding of how diseases cause distinct states of these cell types, this would be contrary to our overall research goals. The below perspective focuses on a framework for benchmarking foundation models for network biology, which model genes and cell states using data such as epigenomics, transcriptomics, and proteomics. However, analogous considerations are also largely applicable to biological models more broadly, such as those modeling protein structure and DNA sequence grammar.

**Key points for benchmarking foundation models for network biology**

First and foremost, we should be benchmarking on biologically meaningful tasks. For example, cell type annotation and batch integration are important preprocessing steps but not our actual biological question. Furthermore, while projecting the data into 2D plots such as UMAPs and *t*‐SNEs is helpful for visualization, assessments are better performed in the higher dimensional space as reduction to two dimensions may overemphasize the most contrastive layer of information, such as cell type, which distinguishes cells more than subtler biological variables such as disease state or age. Relatedly, in the higher dimensional space, optimizing on getting tight cell type clusters by silhouette score may unintentionally penalize approaches for appropriately capturing diversity in disease state, developmental stage, and other aspects of information in the data, as opposed to alternative metrics that focus more on pure separability. Additionally, while pretraining objectives are meant to promote generalizable learning of the informational domain, improved performance on the pretraining objective may not directly correlate with performance on downstream tasks. For example, performance on a masked learning objective may improve with the reduction of the number of genes modeled due to reducing the possible search space, but this may damage the model’s ability to perform biologically meaningful predictions that would benefit from richer information of the gene network state. Overall, the models should instead be evaluated on their ability to make biologically meaningful predictions at both the gene and cell level, such as predicting central regulators within defined gene networks or predicting genes that shift cells between healthy and disease states.

We should also be considering how the “ground truth” was established in what we define as gold standards. For example, it may be clear whether we sampled the cells from a patient who was healthy or affected by a disease diagnosis when we benchmark for predicting disease status. However, the ground truth may not be as clear when benchmarking for predicting cell type labels that were originally annotated from alternative computational methods. If we optimize the models’ abilities to recover, for example, cell clusters derived by linear methods, we will select for models that replicate these prior approaches rather than pushing the boundaries of our knowledge.

Additionally, we should be ensuring generalizability of models rather than overweighting of cell types or diseases that are artificially overrepresented in the scientific literature. For example, immortalized cell lines are easier to assay and peripheral blood may be more accessible to sample from patients, but we want to optimize for models that have a generalizable understanding of biology. We want to ensure we are not optimizing for models that are highly specialized to predicting regulation in K562 cells, for example. This is relevant not only in the training data but also in the tasks we use for benchmarking that guide our evolution of the models.

We should also ensure we are benchmarking on sufficiently challenging tasks including a range of difficulties where alternative simpler approaches have random predictions. Large‐scale foundation models are not going to be required for every task and in many cases simpler approaches will be sufficient. However, as models are scaled up, we may observe emergent capabilities [21], including unpredictable achievement of previously elusive tasks or gaining unexpected knowledge of biological mechanisms relative to the input information. We should therefore be including benchmarks that emphasize to us where these models can really push the boundaries of our predictions.

Furthermore, we should be evaluating performance on a diverse array of tasks to confirm the models have gained generalized knowledge. We should be including discriminative tasks as well as generative tasks. Given the physical constraints of biological systems, it is especially important to ensure the generated output is biologically possible in the real‐world setting.

We should also be employing principled approaches to hyperparameter tuning, which involves optimizing the settings that control the model learning process. The optimal learning hyperparameters are known to be highly specific to the model, data, and task at hand and can make a large difference in the predictive accuracy of the models, ranging from the model learning nothing at all to having near perfect accuracy. We should be ensuring equitable comparisons by standardizing the size of the ranges within which hyperparameters are tuned and exploring the search space with an equivalent number of trials for each model. Furthermore, as with all machine learning methods, separate datasets should be used for training, validation (evaluating model optimizations), and testing of the final optimized model to confirm generalizability. For example, because single‐cell measurements can be highly similar within an individual, datasets for cell‐level tasks are best separated by study, but at the very least by individual patient or cell line from which they were derived.

Finally, we should be designing benchmarks that demonstrate the models’ ability to enable novel discoveries. Prior knowledge may boost predictions in tasks where information is already in the scientific literature, as large models can be very effective at memorizing large‐scale data and returning that information. However, we want to ensure we are evolving models that are not just useful for querying and summarizing prior knowledge but really pushing the boundaries of our knowledge to accelerate novel discoveries. Accomplishing this may require generating general datasets that are held‐out from the literature for the purpose of being used to evaluate a diverse range of downstream tasks and/or experimentally validating that novel predictions are biologically true.

Closely tied to the choice of benchmarks we employ computationally is the ground‐truth experimental data we use to both train and evaluate the models. Thus far, the areas in which data have been generated in the field of biology have been driven to a great extent by feasibility, individual labs’ interests, and funding support so do not represent a truly unbiased random sampling of biological states. For example, much of publicly available large‐scale perturbational datasets are from immortalized cell lines with high mutational burdens that may not fully recapitulate the effects of these perturbations in more natural cell states. However, in the future, large‐scale data may be generated with the explicit goal of obtaining a broad, balanced survey of biological states for training artificial intelligence models to have a generalizable understanding of biology and evaluating the generalizability of their knowledge. Particularly, because gene networks are highly context‐specific, broad perturbational data in cells from a diverse range of states that more closely represent natural development and disease will be highly valuable. For human cells, this may be most currently feasible in induced pluripotent stem cell‐derived cells representing a broad survey of human cell types. Efforts may also be directed at filling knowledge gaps in the literature, such as underrepresented developmental stages including pediatric populations, understudied organ systems or diseases, or time series data to understand the temporal hierarchy of gene network regulation.

By following these principles, we can design a diverse panel of biologically meaningful benchmarking tasks that will promote the evolution of models toward our ultimate goal as a field of making novel biological insights. We can engage in collaborative efforts to generate biological data that will be highly valuable in pushing the knowledge of these models to the next level and develop closed‐loop approaches to synchronously innovate in the computational and experimental domains. As we learn from each model’s unique approach to addressing challenges in network biology, we can determine the best path forward to rapidly evolve our model architectures and training methodologies as a field to generate models with a fundamental understanding of biological systems to accelerate future discoveries.

## AUTHOR CONTRIBUTIONS

**Christina V. Theodoris**: Writing – original draft; writing – review & editing.

## CONFLICT OF INTEREST STATEMENT

None.
